# Postchemoradiotherapy Neutrophil-to-Lymphocyte Ratio Predicts Distant Metastasis and Survival Results in Locally Advanced Pancreatic Cancers

**DOI:** 10.1155/2022/7473649

**Published:** 2022-01-31

**Authors:** Erkan Topkan, Ugur Selek, Veysel Haksoyler, Ahmet Kucuk, Nulifer Kilic Durankus, Duygu Sezen, Yasemin Bolukbasi, Berrin Pehlivan

**Affiliations:** ^1^Department of Radiation Oncology, Baskent University Medical Faculty, Adana, Turkey; ^2^Department of Radiation Oncology, Koc University Schoolof Medicine, Istanbul, Turkey; ^3^The University of Texas MD Anderson Cancer Center, Division of Radiation Oncology, Houston, TX 77030, USA; ^4^Clinics of Medical Oncology, Medline Hospital, Adana, Turkey; ^5^Clinic of Radiation Oncology, Mersin Education and Research Hospital, Mersin, Turkey; ^6^Department of Radiation Oncology, Bahcesehir University, Istanbul, Turkey

## Abstract

**Materials and Methods:**

Our retrospective research included a sum of 126 LAPAC patients who received CCRT. The NLR was calculated for each patient based on the complete blood count test results obtained on the last day of the CCRT. The availability of optimal cutoff(s) that might dichotomize the whole cohort into two groups with significantly different clinical outcomes was searched using receiver operating characteristic (ROC) curve analysis. Primary and secondary endpoints were the potential association between the post-CCRT NLR measures and distant metastasis-free survival (DMFS) and overall survival (OS) outcomes.

**Results:**

The median follow-up duration was 14.7 months (range: 2.4–94.5). The median and 3-year OS and DMFS rates for the whole group were 15.3 months (95% confidence interval: 12.4–18.2) and 14.5%, and 8.7 months (95% CI: 6.7–10.7) and 6.3% separately. The ROC curve analysis findings separated the patients into two groups on a rounded NLR cutoff of 3.1 (area under the curve (AUC): 75.4%; sensitivity: 74.2%; specificity: 73.9%) for OS and DMFS: NLR <3.1 (*N* = 62) and NLR ≥3.1 (*N* = 64), respectively. Comparisons between the NLR groups displayed that the median OS (11.4 vs. 21.4 months; *P* < 0.001) and DMFS (6.0 vs. 16.0 months; *P* < 0.001) lengths were significantly shorter in the NLR ≥3.1 group than its NLR <3.1 counterparts, as well as the 3-year actuarial DM rate (79.7% vs. 50.0%; *P*=0.003). The N1-2 nodal stage, CA 19–9>90 U/mL, and NLR >3.1 were found to be independent predictors of poor prognosis in the multivariate analysis.

**Conclusion:**

The present study found that the posttreatment NLR ≥3.1 was independently linked with a higher risk of DM and subsequent degraded survival outcomes in unresectable LAPAC patients managed with exclusive CCRT.

## 1. Introduction

The prognosis for pancreatic cancer (PAC) is one of the worst of any common solid tumor, with median and 5-year overall survival (OS) rates of only 12 months and 10%, respectively [1–3]. Approximately 30% of all PAC patients have nonmetastatic but locally advanced unresectable disease, namely, locally advanced pancreatic cancers (LAPAC) [4, 5]. There is currently no standard treatment for LAPAC patients; however, chemotherapy (CT), concurrent chemoradiotherapy (CCRT), and induction CT followed by CCRT are the most often utilized alternatives [6]. Despite rigorous therapy, the estimated median survival time of such patients is only around 12 months [7], and 70% of all cases die as a result of extensive distant metastases (DM) [8].

Most neoplastic tissues contain an inflammatory component in their microenvironment, and epidemiological studies have demonstrated that persistent inflammation predisposes to many malignancies [9, 10]. The establishment of the link between cancer and inflammation backs to the eighteenth century based on findings that tumors frequently developed in chronically inflamed tissues and inflammatory cells were abundantly detectable in biopsied tumor samples [11]. The presence of infiltration of white blood cells, polypeptide messengers of inflammation (cytokines such as tumor necrosis factor (TNF), interleukin (IL) 1, IL-6, chemokines such as C-C motif chemokine ligand 2 (CCL2) and C-X-C motif chemokine ligand 8 (CXCL8)), and the eminent activation of the tissue remodeling and angiogenesis processes are all prominent features of cancer-related inflammation [10, 11]. Chronic inflammation has been linked to the development of PAC in several studies [3, 12, 13]. Induction of pancreatitis accelerates the PAC initiation and progression in genetically engineered animal models, and individuals with chronic pancreatitis are known to have a higher risk of developing PAC [14]. Furthermore, a growing body of evidence has shown chronic inflammation as a negative prognosticator for all phases of PAC [14–16].

The neutrophil-to-lymphocyte ratio (NLR) is a simple hematological indicator of systemic immunity and inflammation. A high preoperative NLR predicts a poor outcome for resected early-stage PACs [17, 18]. A low NLR was also an excellent predictor of improved OS and disease-free survival (DFS) in all PAC phases, according to the findings of a recent meta-analysis [19]. According to recent research, higher NLR levels in advanced-stage PAC patients were independently linked to a poor prognosis before and after treatment [20–22]. Furthermore, other studies remarked that elevated levels of pretreatment NLR and the systemic inflammation response index (SIRI) independently predicted poor OS and progression-free survival (PFS) in LAPACs treated with definitive CCRT [3, 23]. We undertook this retrospective analysis to evaluate the link between post-CCRT NLR levels and the prevalence of DM in LAPAC patients treated with CCRT because approximately 70% of all LAPAC patients die from DM and there is a paucity of similar studies.

## 2. Patients and Methods

### 2.1. Study Population

We retrospectively searched the institutional records of LAPAC patients who underwent CCRT Baskent University Medical Faculty's Department of Radiation Oncology between January 2007 and December 2019. Abdominal magnetic resonance imaging (MRI), MR cholangiopancreatography (MRCP), thoracic computed tomography (CT), and brain MRI were used to standardize the staging of all patients. Each patient was also subjected to 18F-fluorodeoxyglucose (FDG) positron emission tomography (PET) CT to improve avoidance of conceivable systemic metastases. Given the lack of reliable guidelines and the comparatively low sensitivity and specificity rates of the currently available CT, MRG, and PET-CT scanning modalities for accurate definition of the involved lymph nodes (N1-2), we determined that any lymph node was involved by combining the commonly used CT (10 mm in short axis) and FDG-PET (standard uptake value >2.5 regardless of nodal size) findings. To be eligible for this review analysis, patients had to satisfy the following additional requirements: age ≥18, Eastern Cooperative Oncology Group (ECOG) performance status 0–1, proven adenocarcinoma histology, clinical T4 tumor (tumor involves celiac axis and/or superior mesenteric artery) according to American Joint Cancer Committee TNM (tumor-node-metastasis) staging system, have completed the scheduled CCRT, available chemotherapy and radiotherapy details, and available follow-up records.

### 2.2. Permissions, Consents, and Ethics

The institutional review board of Baskent University Medical Faculty authorized the research protocol before collecting any patient information. Each participant supplied signed informed permission, either directly or through legislatively appointed deputies, for the collection and analysis of blood samples, pathologic specimens, as well as the publication of results.

### 2.3. Treatment Protocol

All patients underwent radical CCRT with a dosage of 45 Gy RT (1.8 Gy/fraction, 5 days/week, for 5 weeks) that exclusively covered the primary LAPAC site and involved lymph nodes, with elective nodal irradiation being not permitted per our institutional standards. All patients received continuous infusions of 5-fluorouracil (225 mg/m2/day concurrent with RT), followed by 2 to 6 courses of maintenance gemcitabine (1000 mg/m2 intravenously on days 1 and 8 every 21 days).

### 2.4. Neutrophil-to-Lymphocyte Ratio

NLR was calculated for each patient by using the complete blood count test information obtained on the last day of the CCRT course: NLR = neutrophils (N)/lymphocytes (N).

### 2.5. Treatment Response Assessment and Follow-Up

Following completion of the CCRT course, all patients were scheduled for three-monthly (first two years), six-monthly (third to fifth years), and yearly response evaluations. In accordance with EORTC 1999 recommendations, the response was assessed using PET/CT and abdominal MRI scans, as well as complete blood count and biochemistry tests, and serum CA 19–9 measurements. In patients who showed a complete metabolic response on PET-CT scans at any moment, the MRI and chest X-ray scans comprised the chosen diagnostic imaging tools for follow-up evaluations. Additional restaging tools were utilized as necessitated.

### 2.6. Statistical Analysis

Our primary endpoint was DM-free survival (DMFS: interim between the last day of CCRT and the date of DM), whereas the OS (interim between the last day of CCRT and the date of death) comprised the secondary endpoint. Continuous variables were represented by the medians and ranges, whereas categorical variables were described via percentage frequency distributions. The receiver operating characteristic (ROC) curve analysis was used in order to find the accessibility of post-CCRT NLR cutoff (s) that may stratify the research population into two fundamentally distinct OS, DMFS, and PFS outcome groups. The https://www.sealedenvelope.com/power/binary-superiority/ software was utilized to calculate and ensure that the sample size was large enough to reveal at least a 30% survival difference between the two post-CCRT NLR groups with a statistical significance level alpha of 5% (0.05) and adequate power of 0.8 (80%), indicating at least a total of 96 patients (48 patients per group). Chi-square test, Student's *t*-test, Fisher's exact test, or Spearman correlations were carried out for intergroup comparisons, as appropriate. Kaplan–Meier curve estimates and log-rank tests were used to unveil the potential impact of various risk factors on the OS and DMFS outcomes. We appraised the possible interactions between covariates and survival results by using the multivariate Cox proportional hazard model. Any two-sided *P* value <0.05 was considered statistically significant for intergroup comparisons.

## 3. Results

A total of 209 unresectable LAPAC patients who received radiotherapy at our facility were identified. However, only 126 of those individuals were eligible since 83 were excluded from the analysis owing to receiving induction chemotherapy (*N* = 69), self-refusal of concurrent chemotherapy (*N* = 9), and lack of post-CCRT NLR measurements. Baseline patient and disease characteristics are outlined in Table 1. The median age was 58 years (range: 26–79 years), with 34 (27%) of them being elderly patients according to the frequently referenced cutoff of 70 years of age. Male gender (77%), pancreatic head tumor primary (75%), and N1-2 disease status represented the other most prevalent features in the entire research group. Additionally, according to the benchmark Charité Onkologie 001 (CONKO-001) randomized trial's critical threshold for CA 19–9, 72 (60%) patients had CA 19–9 measures ≥90 U/mL [24].

The median follow-up was 14.7 months (range: 2.4–94.5) for the whole study population. At the time of the present analysis, 41 (32.5%), 21 (16.7%), and 35 (27.7%) patients were still alive, progression-free, and DM-free, respectively. The leading cause of mortality was DMs, which accounted for 80 (94.1%) of all 85 death reports, followed by 4 (4.7%) and 1 (1.2%) cases of deaths due to uncontrolled primary disease and chemotherapy toxicity, respectively. Respective median and 3-year OS and DMFS rates were 15.3 months (95% confidence interval: 12.4–18.2) and 14.5%, and 8.7 months (95% CI: 6.7–10.7) and 6.3% separately. All patients underwent evaluations for conversion surgery 6-weeks after completion of CCRT, where the procedure was judged viable for just 12 (9.5.0%) of the patients, with R0 resection being attainable in 7 (5.6%) cases.

**Figure 1:**
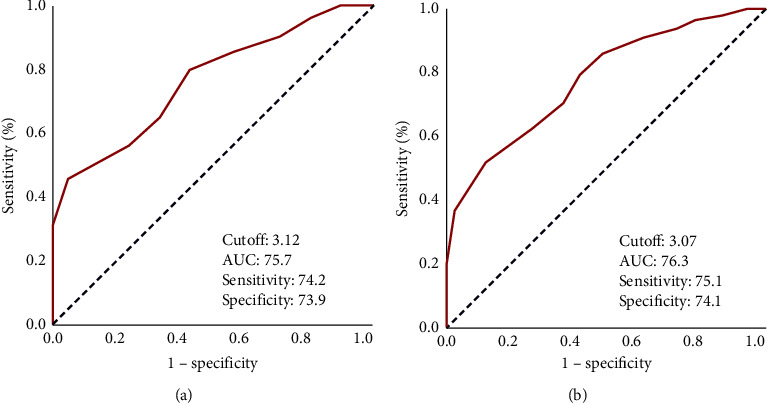
Receiver operating characteristic curve analysis results examining the relationship between the posttreatment neutrophil-to-lymphocyte ratio and survival outcomes. (a) Overall survival and (b) distant metastasis-free survival.

**Figure 2:**
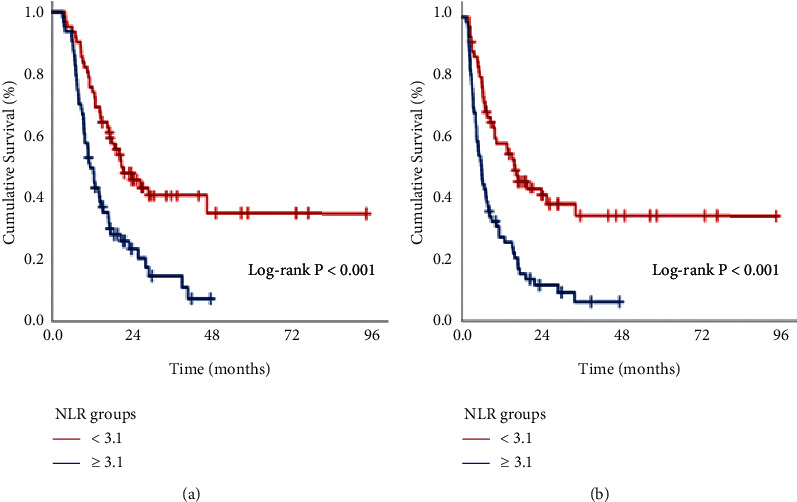
Comparative survival outcomes per posttreatment neutrophil-to-lymphocyte ratio. (a) Overall survival and (b) distant metastasis-free survival.

In order to investigate the presence of cutoffs for meaningful relationships between the post-CCRT NLR values and the OS and DMFS results, we have carried out ROC curve analyses, which determined the ideal cutoffs at 3.12 (area under the curve (AUC): 75.7%; sensitivity: 74.2%; specificity 73.9%) and 3.07 for OS and (AUC: 76.3%; sensitivity: 75.4%; specificity: 74.1%) and DMFS, respectively (Figure 1). Because the two cutoffs were very close, we picked 3.1 as the cutoff that grouped patients into two bunches: low-NLR (L-NLR: NLR <3.1) and high-NLR (H-NLR: NLR ≥3.1) bunches, individually. As depicted in Table 1, the baseline characteristics were virtually similarly distributed across the two NLR bunches with no notable differences except for the fact that the CA-19-9 >90 patients were significantly more common in the NLR ≥3.1 than the NLR <3.1 bunch (67.2% versus 46.8%; *P*=0.002). Comparative Kaplan–Meier analyses exhibited that the NLR ≥3.1 group had significantly shorter median DMFS (6.0 vs. 16.0 months; *P* < 0.001) and OS (11.4 vs. 21.4 months; *P* < 0.001) durations than the NLR <3.1 group (Figure 2). The 3-year DMFS (34.5% vs. 6.3%) and OS (40.6% versus 14.5%) rates were likewise inferior in the NLR ≥3.1 group, as well as the 3-year actuarial DM rate (79.7% vs. 50.0%; *P*=0.003).

The results of the univariate analyses showed that N1-2 nodal stage (versus N0), CA 19–9>90 U/mL (versus ≤90 U/mL), and NLR ≥3.1 (versus NLR <3.1) were the factors predicting inferior median DMSF (*P* < 0 : 05, for each) and OS (*P* < 0 : 05, for each) outcomes, separately (Table 2). The results of multivariate analyses confirmed that all three factors had separate independent prognostic significance on DMSF (*P* < 0 : 05, for each) and OS (*P* < 0 : 05, for each) results in their own ways (Table 2). Further investigation into the possibility of a probable link between pre- and posttreatment NLR values revealed that the pre-CCRT NLR was significantly higher (3.54 vs. 2.18; *P* < 0.0014) in the post-CCRT NLR ≥3.1 group than in the NLR <3.1 peers. Affirming this finding, Spearman correlation analysis showed a strong connection between pre- and post-CCRT high NLR values (*r*_*s*_ = 0.87; *P* < 0.0016).

We have also conducted analyses to determine the pre-CCRT cutoffs for NLR measures as pre- and post-CCRT values were strongly correlated. Our search yielded the optimal cutoffs at 2.94 (AUC: 66.2%; sensitivity: 67.9%; specificity: 65.1%) for DMFS and 2.87 for OS (AUC: 64.1%; sensitivity: 65.6%; specificity: 62.3%), which separated patients into two groups with substantially different survival outcomes. Accordingly, we selected the rounded 2.9 as the common cutoff for further comparative analysis. Group 1: NLR values pre-CCRT NLR had lower discriminating value for survival outcomes than the post-CCRT NLR. Kaplan–Meir survival analysis results exhibited that the NLR ≥2.9 group had poorer median DMFS (7.4 vs. 11.9 months; *P* < 0.012) and OS (13.1 vs. 19.3 months; *P* < 0.009) outcomes compared to its NLR <2.9 counterpart, separately. As revealed from the *P* values pre-CCRT NLR had lower discriminating value for survival outcomes than the post-CCRT NLR.

## 4. Discussion

We attempted to clarify the prognostic validity of post-CCRT NLR measurements in this retrospective cohort study of 126 unresectable LAPAC patients treated with radical CCRT. Our findings unveiled that the patients with post-CCRT NLR ≥3.1 had an expanded 3-year risk of DM (79.7% vs. 50.0%; *P*=0.003), as well as diminished DMFS (34.5% vs. 6.3%) and OS (40.6% versus 14.5%) results as opposed to those patients with NLR <3.1. These findings imply that persistent systemic inflammation following CCRT might be linked to the existence of pretreatment occult metastases and resistance to currently available systemic therapies.

Our current findings, like most prior studies, confirmed the prognostic merit of the presence of involved lymph nodes (stage N1-2) and high CA19-9 (>90 U/m/L) status as signatures of worse survival outcomes in LAPAC patients. However, our study appears to be the first to examine the prognostic usefulness of post-CCRT NLR and the probable connection between the pre-CCRT and post-CCRT NLR levels in unresectable LAPAC patients treated with definitive CCRT. We demonstrated a clear connection between the pretreatment and posttreatment high NLR values, indicating a highly aggressive, persistently inflamed, and immune-suppressed LAPC phenotype that cannot be meaningfully changed by CCRT. Intimating a solid link between a persistent and exacerbated inflammation and impaired systemic immunity status and poor clinical outcomes, we further discovered that post-CCRT NLR ≥3.1 values were associated with significantly shorter OS (*P* < 0.001) and DMFS (*P* < 0.001) results. Owing to the unavailability of comparator LAPAC investigations, it is formidable for us to comment decisively on our novel findings. However, the exhibition of an independent link between the poorer survival outcomes and NLR >3.1 values seems to accord well with the historic results reported for postoperative PAC patients undergoing systemic chemotherapy, immune checkpoint inhibitor therapy, or preoperative chemoradiotherapy for rectal and gastric cancers [25–29]. According to Pu et al. [25], high levels of postoperative NLR were associated with significantly poorer OS and relapse-free survival in a cohort of 97 patients treated with radical surgery. In a recent study comprising LAPAC and metastatic PAC patients, Shang et al. demonstrated that continuously elevating NLR values after immune checkpoint inhibitors was associated with poorer outcomes compared to their counterparts with reducing NLR values after therapy [26]. Cha et al. recently reported that persistently high NLR (NLR >3) values were linked to a greater risk of rectal cancer recurrence and a lower progression-free survival rate following induction chemoradiotherapy [27]. Likewise, Sung et al. announced that the persistently elevated post-CCRT NLR measures were substantially linked with increased DM rates in patients with locally advanced rectal cancer who received preoperative CCRT followed by curative total mesorectal excision and adjuvant chemotherapy [28]. Similar findings were reported also in 734 gastric cancer patients treated with total or subtotal gastrectomy who had persistently elevating NLR values after the surgical procedure [29]. Taken together, the results of all preceding studies and those provided here for LAPAC patients strongly propose that the posttreatment high levels of NLR may be utilized as a reliable indicator of poor treatment outcomes irrespective of the tumor type, tumor stage, and choice of radical treatment.

We chose the DMSF and the actual DM rates as the two surrogate measures to evaluate the prognostic potential of post-CCRT NLR values. This decision was largely influenced by the evidence indicating that two-thirds of all LAPAC-related deaths are directly ascribed to the disease's inevitable widespread DM development, which frequently defies all known rescue efforts [8]. We observed DMs in 91 (72.7%) of 126 patients in the entire research sample, lending credence to these findings. Furthermore, indicating a solid link between exacerbated inflammation and elevated rates of DMs, the 3-year actuarial DM rate in the NLR ≥3.1 group was significantly higher than its NLR <3.1 counterparts (79.7% vs. 50.0%; *P*=0.003). Although the exact association may be more complex, neutrophils are one of the well-known myeloid cell lineages that play prominent roles in tumor genesis, growth, and metastasis. Neutrophils can stimulate tumor invasion by generating and releasing a wide range of proteins, particularly serine proteases, into the extracellular environment, which degrades many extracellular matrix components and removes the barrier to cancer invasion and metastasis [30]. These tumor cell-mobilized neutrophils have been shown to impede natural killer cell functions and facilitate tumor cell survival in the peripheral circulation [31]. Neutrophils can also help tumor cells' extravasation by secreting matrix metalloproteinase-9, which also plays a role in the creation of aberrant vasculature with diminished pericyte and smooth muscle coverage and disrupted interendothelial junctions [32]. Within the premetastatic niche, neutrophils may perform a variety of prometastatic roles, including providing tumor trophic factors, promoting angiogenesis, and suppressing immunity, all of which contribute to tumor survival and proliferation at the metastatic site [30]. As a result, an elevated NLR value may indicate an immune-suppressed premetastatic condition, as neutrophils play key roles in all phases of the metastatic process, which may manifest clinically as the emergence of early and overt metastasis, as seen here.

Admittedly, the intricate link between the seventh cancer hallmark, inflammation, and tumor metastasis traces back to Virchow's pioneering hypothesis in the nineteenth century [11]. The precise mechanism(s) underpinning the causal reciprocity between a high NLR measure and dramatically diminished clinical results in LAPAC patients has yet to be determined. Nonetheless, by assessing the unique immune and inflammatory functions of the neutrophil and lymphocyte constituents of the NLR, as well as the basic knowledge implying that immune and inflammatory cells account for nearly half of the overall tumor burden of the LAPACs [33], it is possible to propose some wise comments. Lymphocyte counts and functions are major determinants of the host's anti-cancer immune response to tumor cells and hence clinical outcomes following different oncological treatments. Accordingly, while normal or increased peripheral lymphocyte counts indicate an intact immune response, any decrease in lymphocyte counts during exacerbated systemic inflammation, on the other hand, signals severely compromised immune surveillance and host defense mechanisms with sequent poor outcomes [34]. The neutrophils bear critical roles in the incitement of inflammatory chemokines and cytokines, as well as in the promotion of cancer cell proliferation, neoangiogenesis, tumor invasiveness, metastatic potential, and induction of resistance to chemotherapy and radiotherapy [35]. Additionally, granulocytic myeloid-derived suppressor cells (G-MDSCs), a subpopulation of circulating neutrophils, may suppress antitumor immune responses by promoting tumor growth and invasiveness via suppressing the T-cell proliferation and activation [34, 35]. As a result, increased neutrophil counts imply a heavier tumor load and an aggravated inflammatory state, both of which reduce the effectiveness of any treatment. Taken together, elevated neutrophil and concomitantly reduced lymphocyte counts indicate a severely compromised anti-cancer immunity and extremely aggravated systemic inflammation, which enables resistance to treatments and early DMs. Supporting the significantly increased DM and related fatalities in the NLR ≥3.1 group observed here, Albrengues et al. demonstrated that the persistent inflammation was functioning as a facilitator of metastasis [36]. Moreover, the authors claimed that chronic inflammation was able to reawaken the dormant cancer cells by recruiting neutrophils to metastatic sites, where they remodel the extracellular matrix and stimulate cancer cell proliferation. Further support comes from the studies demonstrating that the chronic inflammation may mobilize the myeloid cells from the bone marrow and recruit them to the liver or lung, where they establish a niche supportive of PAC metastasis during the early steps of carcinogenesis [37–39], which is currently impossible to detect with current staging tools. Despite recognizing the need for affirmative studies, it is still rational to anticipate that high NLR values indicate an inferior prognosis in radically treated LAPACs since they reflect the hosts' weakened immune and exacerbated systemic inflammation condition simultaneously. Several facts hampered our present investigation. First and foremost, it was single-center retrospective research with small sample size. Second, the ideal NLR cutoff value was determined to be 3.1, which reflected only a single time point measure. As a result, given the widely fluctuating nature of the NLR at various time points over the adjuvant chemotherapy time frame, the present NLR cutoff may not reflect the ideal cutoff that separates LAPAC patients into two best-fit groups with substantially different clinical outcomes. Third, we did not investigate if NLR might be utilized in conjunction with other prometastatic, apoptotic, phagocytosis, and inflammation markers to uncover a putative relationship between the prolonged systemic inflammatory response and early systemic metastases in the NLR3.1 group. And fourth, because varied rescue therapy choices may accidentally bias the results in favor of one group, the current findings are inconclusive whether they can be generalized to all unresectable LAPAC patients treated with radical CCRT. Therefore, well-designed large-scale studies focusing on these critical aspects may provide useful information to guide the customized therapy of SIRI >3.1 LAPAC patients in terms of both upfront and adjuvant anticancer therapies.

## 5. Conclusion

In conclusion, the current findings demonstrated that the posttreatment NLR ≥3.1, a simple and inexpensive biomarker of the chronic systemic inflammatory response, was independently associated with increased risk of DM and subsequent depreciated survival outcomes in unresectable LAPAC patients treated with exclusive CCRT, highlighting the urgent need for the development of novel and more powerful systemic treatments.

## Figures and Tables

**Table 1:** Baseline patient and disease characteristics.

Characteristics	All patients (*N* = 126)	NLR <3.1 (*N* = 62)	NLR ≥3.1 (*N* = 64)	*P* value
Median age, years (range)	58 (26–79)	59 (26–79)	57 (34–78)	0.92
Age groups, *N* (%)				0.81
** **<70	92 (73.0)	45 (72.6)	47 (73.4)	
** **≥70	34 (27.0)	17 (27.4)	17 (26.6)	
Gender, *N* (%)				0.79
** **Female	29 (23.0)	14 (22.6)	15 (23.4)	
** **Male	97 (77.0)	48 (774)	49(76.6)	
ECOG performance, *N* (%)				0.42
** **0	41 (32.5)	21 (33.9)	20 (31.3)	
** **1	85 (67.5)	41 (66.1)	44 (68.7)	
Tumor location, *N* (%)				0.76
** **Head	94 (74.6)	45 (72.6)	49 (76.6)	
** **Body/tail	32 (25.4)	17 (37.4)	15 (23.4)	
Median tumor size, mm (range)	37 (26–79)	35 (26–74)	39 (28–79)	0.28
Tumor size group, *N* (%)				0.19
** **<37 mm	60 (47.6)	32 (51.6)	28 (43.8)	
** **≥37 mm	66 (52.4)	30 (48.4)	36 (56.2)	
N-stage, *N* (%)				0.23
** **0	61 (48.4)	32 (51.6)	29 (45.3)	
** **1–2	65 (51.6)	30 (48.4)	35 (54.7)	
CA 19–9, *N* (%)				0.002
** **≤90 U/m/L	54 (42.9)	33 (53.2)	21 (32.8)	
** **>90U/m/L	72 (57.1)	29 (46.8)	43 (67.2)	
Median pre-CCRT NLR	2.71 (1.5–5.7)	2.18 (1.5–4.1)	3.54 (2.3–5.7)	0.0014)

*Abbreviations.* NLR: neutrophil-to-lymphocyte ratio; ECOG: Eastern Cooperative Oncology Group; N-stage: nodal stage; CA 19–9: cancer antigen 19–9; CCRT: concurrent chemoradiotherapy.

**Table 2:** Outcomes of uni- and multivariate analysis.

Characteristics	Overall survival			Distant metastasis-free survival		
	Univariate	Multivariate	HR	Univariate	Multivariate	HR
	*P* value	*P* value		*P* value	*P* value	
Age group (<70 vs. ≥70 years)	0.93	—	—	0.78	—	—
Gender (female vs. male)	0.79	—	—	0.72	—	—
ECOG (0 vs.1)	0.90	—	—	0.83	—	—
Tumor location (H vs. B/T)	0.64	—	—	0.76	—	—
Tumor size (<vs. ≥3.7 mm)	0.41	—	—	0.48	—	—
LN status (N0 vs. N1-2)	0.007	0.005	1.72	<0.001	<0.001	2.16
CA 19–9 (<vs. ≥90 U/mL)	0.001	0.002	1.94	0.001	0.001	2.04
NLR (<vs. ≥3.1)	<0.001	<0.001	3.72	<0.001	<0.001	5.68

*Abbreviations.* HR: hazard ratio; ECOG: Eastern Cooperative Oncology Group; H: head; B/H: body/tail; NX: nodal stage x; CA 19–9: cancer antigen 19–9; NLR: neutrophil-to-lymphocyte ratio.

## Data Availability

Data cannot be shared publicly because the data are owned and saved by Baskent University Medical Faculty. Data are available from the Baskent University Radiation Oncology Institutional Data Access/Ethics Committee (contact via Baskent University Ethics Committee) for researchers who meet the criteria for access to confidential data: contact address, adanabaskent@baskent.edu.tr.
